# Further adventures of the perovskite family

**DOI:** 10.1107/S2052252522008673

**Published:** 2022-09-01

**Authors:** Anthony E. Phillips

**Affiliations:** a School of Physical and Chemical Sciences, Queen Mary University of London, London E1 4NS, United Kingdom

**Keywords:** metal-free perovskite, polymorphism, disorder, ionic crystals

## Abstract

The perovskites are an intensely studied class of materials, with a breadth of possible compositions made even wider by the possibility of incorporating molecular ions. Here the context is discussed of a newly reported metal-free perovskite with the H_3_O^+^ ion on the *B* site.

The well known perovskite structure consists of ‘*B*-site’ nodes connected by ‘*X*-site’ linkers into a cubic network with an ‘*A*-site’ ion sitting at each interstice [Fig. 1 ▸(*a*)]. It is justly celebrated among crystallographers because it combines an extremely stable *topology* with a rather precarious *geometry* (Salje, 1989 ▸). In other words, although the connectivity just described is extremely common, its most symmetrical realization, the 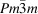 aristotype structure, is comparatively rare. Even the mineral perovskite itself, CaTiO_3_, is orthorhombic under ambient conditions, only transforming to the cubic phase (via a tetragonal intermediate) above 1635 K (Yashima & Ali, 2009 ▸).

This observation is more than simply a crystallographic curiosity, since it is precisely this instability that causes these materials to respond readily to external stimuli. This in turn gives functionality including dielectric, piezoelectric and ferroelectric behaviour (Bhalla *et al.*, 2000 ▸), and oxide, proton and electronic conduction (Kreuer, 2003 ▸; Richter *et al.*, 2009 ▸). Entire communities of scientists have grown up around each of these properties, working on exploiting these materials’ rich behaviour for functional applications (Tilley, 2016 ▸).

Group-theoretical analysis has contributed significantly to understanding the possible distortions from the aristotype symmetry (Salje, 1976 ▸; Howard & Stokes, 1998 ▸, 2005 ▸). In fact, the perovskite family was an important historical motivation for developing this sort of analysis. Perovskites were a very early example of the work of Bärnighausen on the trees that now bear his name (Fig. 2 ▸). Even the very words ‘aristotype’ and ‘hettotype’ were coined by Megaw (1973 ▸) to describe the perovskite structure.

Importantly, the propensity to structural distortion is genuinely a property of the perovskite structure, rather than the mineral perovskite itself. It is straightforward to replace – either outright or as a solid solution – the ion at the *A*, *B* or *X* sites, producing a large family of materials with varying chemistry and, therefore, different active distortion modes. Thus the *structural* flexibility of this family of materials is accompanied by a corresponding *compositional* flexibility.

In particular, none of the *A*, *B* or *X* ions need be a single atom. It has been well known for at least fifty years that (poly)methylammonium ions can be inserted on the *A* site of halide-bridged perovskites (Weber, 1978*a* ▸,*b* ▸). At around the same time, materials with longer, bidentate *X*-site ions such as formate (HCO_2_^−^) were first reported (Sletten & Jensen, 1973 ▸), although these were not initially described as perovskites. Thus, while the term ‘organic–inorganic’ or ‘hybrid’ perovskites originally referred specifically to materials where the *A*-site ion was organic (Mitzi, 1999 ▸), it was soon extended to include organic *X* linkers such as cyanide, formate or dicyanamide (Li *et al.*, 2017 ▸). These two developments naturally complement one another: as the *X*-site linker becomes longer, there is more space for a larger *A*-site cation [Figs. 1 ▸(*b*), 1 ▸(*c*)]. This opens very powerful new possibilities for crystal engineering: we can change not only the size of these ions, but also their shape, chemistry (*e.g.* replacing a metal by an organic ion) and physics (*e.g.* incorporating directional hydrogen bonding as well as isotropic Coulomb and van der Waals interactions).

Hybrid perovskites take after their inorganic counterparts in each of the ways described above. They undergo complex structural distortions, in several cases having incommensurately modulated phases (Fütterer *et al.*, 1995 ▸; Canadillas-Delgado *et al.*, 2019 ▸). They have important applications – most spectacularly in solar cells (Kojima *et al.*, 2009 ▸), but also as ferroelectrics (Guo *et al.*, 2010 ▸) and multiferroics (Jain *et al.*, 2009 ▸). And theoretical analysis has revealed the structural possibilities that arise from the new degrees of freedom afforded by polyatomic linkers (Boström *et al.*, 2016 ▸, 2018 ▸).

Both inorganic and hybrid perovskites are flexible in yet another sense: small variations in ionic radius or stoichiometry will often produce related structures that are informally considered under the wider perovskite banner. Common examples of such structures include the ‘hexagonal perov­skites’, where *BX*_6_ octahedra, rather than sharing vertices, form face-sharing columns (Lander, 1951 ▸; You *et al.*, 2017 ▸); and layered structures such as the Dion–Jacobson, Ruddlesden–Popper and Aurivillius phases (Schaak & Mallouk, 2002 ▸; Saparov & Mitzi, 2016 ▸). As a result, the boundaries of the perovskite family are, appropriately, themselves rather flexible – even if we have not quite yet reached the stage of ‘radical perovskite anarchy’ (Palgrave, 2019 ▸).

The discussion above of hybrid perovskites has conspicuously neglected the case of molecular ions on the *B* site. It turns out that this is no exception, accommodating polyatomic ions as readily as the *A* or *X* sites (Bremner *et al.*, 2002 ▸). This produces yet another new sub-family of *metal-free* perovskites, some of which have impressive ferroelectric properties comparable to their inorganic counterparts (Ye *et al.*, 2018 ▸).

In this issue, Budzianowski *et al.* (2022 ▸) present a new dabco-based, metal-free perovskite that for the first time has not the ammonium ion but the hydronium ion, H_3_O^+^, on the *B* site. Once again, incorporating new chemistry into the perovskite structure has enabled new crystallography to emerge.

Both polymorphs of the new material are intriguing in different ways. The α polymorph, with the true perovskite topology, crystallizes in the chiral space group *P*3_2_21, which, although analogous to the ammonium analogue, is perhaps unexpected given these materials’ simple, highly symmetrical components. The β polymorph is a polar, face-sharing hexagonal perovskite. Its structure is in effect a *commensurate* modulation of a non-polar structure with a cell three times smaller (and the authors go to some length to demonstrate that this simpler model does not adequately describe their data). The structure of both polymorphs is clearly influenced by the varying degrees of disorder of the molecular components, both hydronium and dabco ions.

This work will be important to at least three frontiers in materials chemistry and physics. First, the topic of metal-free perovskites is rapidly evolving into a subfield in its own right (Cui *et al.*, 2022 ▸). Second, substituting polyatomic for monatomic ions is now acknowledged as a way of achieving novel degrees of freedom beyond the specific perovskite family (Boström & Goodwin, 2021 ▸). Third and more generally still, the study of structural disorder in molecular materials such as this one is an important frontier in crystallography. Such disorder may be a desideratum in its own right (Das *et al.*, 2020 ▸) or even harnessed as a crystal engineering tool to direct the formation of particular structures.

After so many years of intense research, one might think that little remains to be discovered about the perovskite structure. But, as Budzianowski, Petřiček and Katrusiak have demonstrated, with a subtle change in chemistry, it retains its power to surprise crystallographers once again.

## Figures and Tables

**Figure 1:**
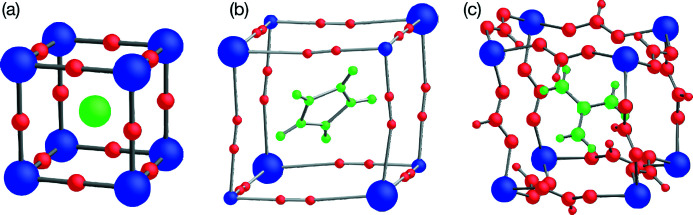
The *A* (green), *B* (blue), and *X* (red) sites in conventional and hybrid perovskites, showing the larger *A*-site ions possible as the length of the *X*-site linker increases: (*a*) monatomic *X* and *A* (Goldschmidt, 1927 ▸); (*b*) diatomic *X* = cyanide, *A* = imidazolium (Zhang *et al.*, 2010 ▸); (*c*) triatomic *X* = formate, *A* = guanidinium (Hu *et al.*, 2009 ▸).

**Figure 2:**
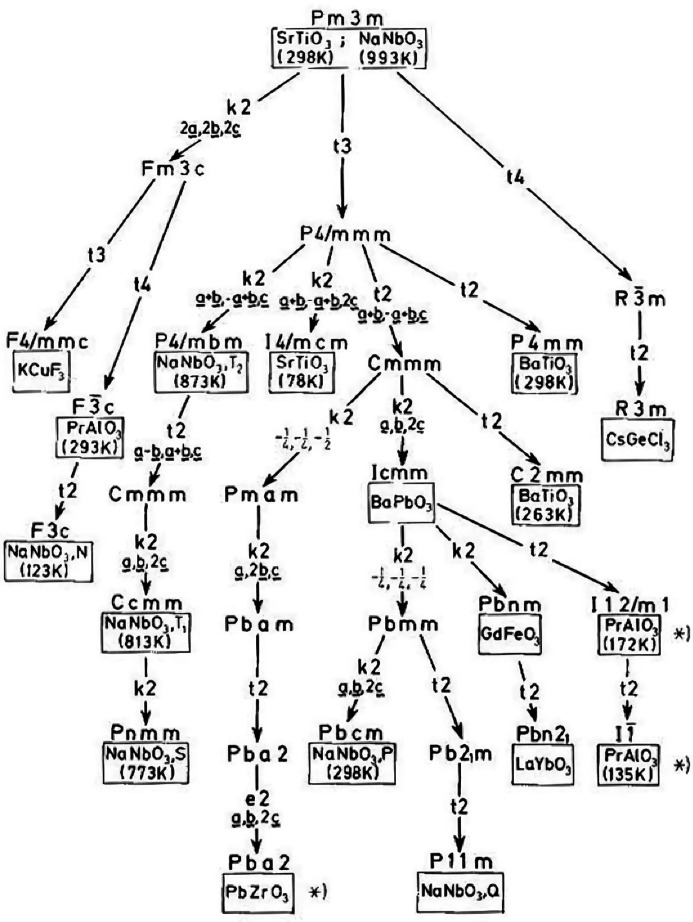
Bärnighausen’s (1975 ▸) original ‘family tree’ of perovskites.
